# Radial Wettable Gradient of Hot Surface to Control Droplets Movement in Directions

**DOI:** 10.1038/srep10067

**Published:** 2015-05-15

**Authors:** Shile Feng, Sijie Wang, Yuanhao Tao, Weifeng Shang, Siyan Deng, Yongmei Zheng, Yongping Hou

**Affiliations:** 1 Key Laboratory of Bio-Inspired Smart Interfacial Science and Technology of Ministry of Education, Beijing Key Laboratory of Bio-inspired Energy Materials and Devices School of Chemistry and Environment Beihang University. Beijing, 100191 (P. R. China)

## Abstract

A radial wettable gradient was fabricated on the surface of graphite plate by a simple one-step anodic oxidation process. It was found that the direction and value of the wettable gradient could be easily controlled by adjusting current and oxidation time gradient. With the increase of surface temperature, droplets on surface not only exhibited the transition of boiling mode, but also showed the controlled radial spreading, evaporation and movement behaviors. These phenomena could be attributed to the cooperation of wettability force, hysteresis force and vapor pressure (Leidenfrost effect). Especially, the controlled radial convergence or divergence of droplets with high velocity were realized on the surfaces with either inside or outside radial gradient, which would have crucial applications in the design of microfluidic devices and the exploration of the biotechnology.

The directional movement behaviors of droplets on a surface with wettable gradient exhibit important applications in the design of microfluidic devices and exploration of the biotechnology^1^,^2^,^3^,^4^. The different features on wettability at the front and rear of the droplet, which introduces the unbalanced force around the periphery of the droplet,^5^ could be achieved via controlling the surface chemical composition and tailoring the surface morphology^6^,^7^,^8^,^9^. Chaudhury and Whitesides first created a wettable gradient surface via chemical deposition and further demonstrated that a droplet ran uphill spontaneously on the inclined surface^8^.Ashutosh Shastry *et al.*^10^ fabricated a surface with gradient wettability resulted from the solid-liquid contact area fraction, on which droplets motion directionally. Ding-Jun Huang *et al.*^11^ described a fabrication process of the surface energy gradient on copper substrate by H_2_O_2_ immersion and fluorination with Teflon, which effectively controlled the change of wettability from superhydrophilic to superhydrophobic ones. On the other hand, liquid motion could be controlled by introducing external stimuli such as light^12^,^13^, heat^14^, vibration^15^ and non-equilibrium noise^16^. Kunihiro Ichimura *et al.*^12^ modified silica plate with a photoisomerizable azobenzene monolayer and the motion of liquids could be manipulated reversibly by photo irradiation. George Karapetsas *et al.*^14^ propelled droplets by a non-uniformly heated substrate. Susan Daniel *et al.*^15^ fabricated a surface of relatively high modulus poly-(dimethylsiloxane) (PDMS) on a glass and the droplets were directionally driven by asymmetric lateral vibration. In the previous studies, the fabrication and controlled methods of gradient surface were difficult to implement and the direction of wettable gradient was simple. Although the controlled radial movement of droplets has more important potential applications^4^, few researches have been done due to the difficult fabrication of radial wettable gradient. Therefore, it remains challenging to fabricate gradient at multi-dimensions (e.g., radial direction of gradient on a surface) achieve the controlled radial movement of droplets via a simple, fast and efficient method. Here, we fabricated a radial wettable gradient surface by a simple one-step anodic oxidation process. By controlling the current gradient and oxidation time, the direction and value of the wettable gradient could be easily adjusted. At ambient temperature, droplets on the wettable gradient surface showed radial spreading, and at high temperature, the radial evaporation or motion of droplets was realized due to the cooperation between the Leidenfrost effect^17^,^18^ and the wettable gradient driving force. These new phenomena would promote the development of microfluidic device and the design of heat exchanger.

## Results

**Figure 1:**
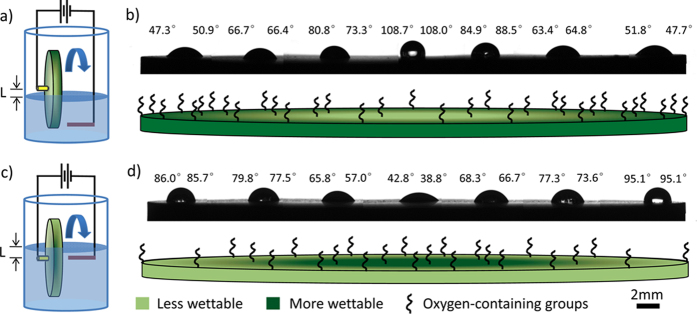
Schematic diagrams of experimental set-up for the fabrication of the wettable gradient and the photographs of the water contact angles. **a**) Experimental set-up. Electrolyte level is below the center. The point close to the center has a shorter oxidation time and lower current density. A radial wettable gradient is fabricated with the direction from center to periphery. **b**) Photographs of the water contact angles along the graphite surface with wettable direction from center to periphery. More oxygen-containing groups are formed on the periphery. **c**) Electrolyte level is above the center, the radial wettable gradient is in opposite direction, from periphery to center. **d**) Photographs of the water contact angles along the graphite surface with wettable gradient direction from periphery to center. More oxygen-containing groups are formed on the central parts.

A circular graphite plate with radial wettable gradient was fabricated by an improved anodic oxidation (see experiment section). As shown in Fig. 1a, when the electrolyte level is below the center for a given distance (*L*), the oxidation time (*t*_rb_) could be described as:


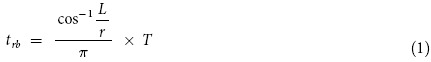


Where *t*_rb_ is the anodic oxidation time at the point away from the center of graphite plate with a distance of *r*. *T* is the rotation period. We plotted the value t_rb_/*T* as a function of the value of *L*/*r*, which is shown in Figure S1a. For given values of *L* and *T*, the time of anodic oxidation (*t*_rb_) is positive correlated with *r*. In addition, because of the position relation of the cathode and anode, a current gradient could be formed during the oxidation process^19^. On the binary collaboration effect above (time gradient and current gradient), the radial wettable gradient is gained with the direction from center to periphery (the point close to the center has a shorter oxidation time and lower current density). For Sample-A (*L* = 3 mm, *T* = 5 s), contact angles (CAs) decrease from ~108.3 ^o^ to ~47.5 ^o^ with the increase of *r*, showing a wettable gradient of ~4.1 ^o^/mm (Fig. 1b). When the electrolyte level is above the center for a given distance (*L*) (Fig. 1c), for each point on the surface of graphite plate, the oxidation time (*t*_ra_) could be described as:


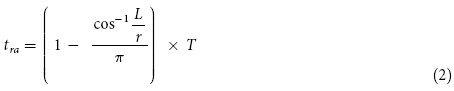


Here *t*_ra_ is the anodic oxidation time at the point away from center of graphite plate with a distance of *r*. For given values of *L* and *T*, the *t*_ra_ could be regarded as a function of the *r* (Figure S1b). Clearly, the *t*_ra_ decreases gradually with the increase of the *r*. Based on the same principle as mentioned above (time gradient and current density gradient), a radial wettable gradient could be fabricated with the direction from periphery to center. For Sample-B (*L* = 3 mm, *T* = 5 s), the CAs could change from ~40.8 ^o^ to ~85.8 ^o^ gradually and the average value of wettable gradient achieves ~3.5 ^o^/mm. We also investigated the influence of two parameters, e.g., *L* and *T* on the wettable gradient. The results indicate that the value of wettable gradient could be adjusted from ~ 2.1 to ~ 4.1 ^o^/mm and the *L* seemed to have a greater influence on the wettable gradient (Figure S2). Clearly, the radial wettable gradient on a circular graphite plate could be easily fabricated by an improved anodic oxidation method and both the direction and value of wettable gradient could be controlled via changing the two parameters, i.e., *L* and *T*.

Energy dispersive spectrometer (EDS) experiment of Sample-A was shown in Figure S3 and Table S1. The ratio of O/C increases gradually from 0.0448 to 0.3467 with the increase of distance away from the center. Due to the fact that the C content can be considered as a constant on the surface of graphite plate, a gradient of O content is formed during the anodic oxidation process and more oxygen-containing surface groups (C–OH, C = O, COOH) are formed on the periphery of graphite plate^19^. In addition, scanning electron microscope (SEM) observations show that the surface is very rough and the change of morphology at different areas is little (Figure S4). It has been recognized that the topographic structures and the surface chemical compositions are crucial to surface wettability. In our experiments, all of the results indicate that the wettable gradient on graphite plate is mainly controlled by the gradient of chemical composition, and the value of wettable gradient is amplified by the roughness of surface (i.e., from hydrophilic to more hydrophilic ones, or from hydrophobic to more hydrophobic ones^20^).

Although a large wettable gradient is already realized (~4.1 ^o^/mm), the water droplets could only exhibit directional spreading at room temperature (shown in Figure S5 and Figure S6). On both surfaces (Sample-A and Sample-B), when a water droplet comes in contact with the surface, it spreads along the wettable gradient immediately and shows a asymmetric shape due to large wettable gradient forces and high hysteresis force^21^.

It is well known that a liquid would levitate on a cushion of its own vapor when it contacts with a hot solid (Leidenfrost effect). Linke *et al.*^17^ used the effect to reduce the hysteresis forces and realized the self-propulsion of droplets on the asymmetric topology surface. Ali Hashmi *et al.*^18^ demonstrated that a small cart could be levitated by Leidenfrost vapor to reduce the Friction. The cart could be propelled not only by gravitational force over a slanted flat surface, but also self-propelled over a ratchet shaped horizontal surface. Here, we also heated the graphite plate to reduce the hysteresis forces to control the motion of droplets. When a liquid droplet is dripped on a surface heated above its boiling point, three typical cases are formed, nucleate boiling (the droplet boils immediately when it contacts the heated surface), gentle film boiling (the droplet floats on its own vapor called Leidenfrost vapor layer without any contact with the surface), and spraying film boiling (both Leidenfrost vapor layer and sputtering tiny droplets). The critical temperature for the formation of Leidenfrost vapor layer is defined as Leidenfrost temperature (*TL*)^22^. Figure S7 shows the negative correlation of the Leidenfrost temperature and CAs at different positions of Sample-A^23^. With the decrease of CAs (Fig. 1b, S7), Leidenfrost temperature increases from ~143.2 ^o^C to ~149.7 ^o^C due to the fact that hydrophobic surface performs more favorable conditions for the formation and growth of bubbles than hydrophilic surface and the generated bubbles are easier to merge to vapor blanket^23^.

**Figure 2:**
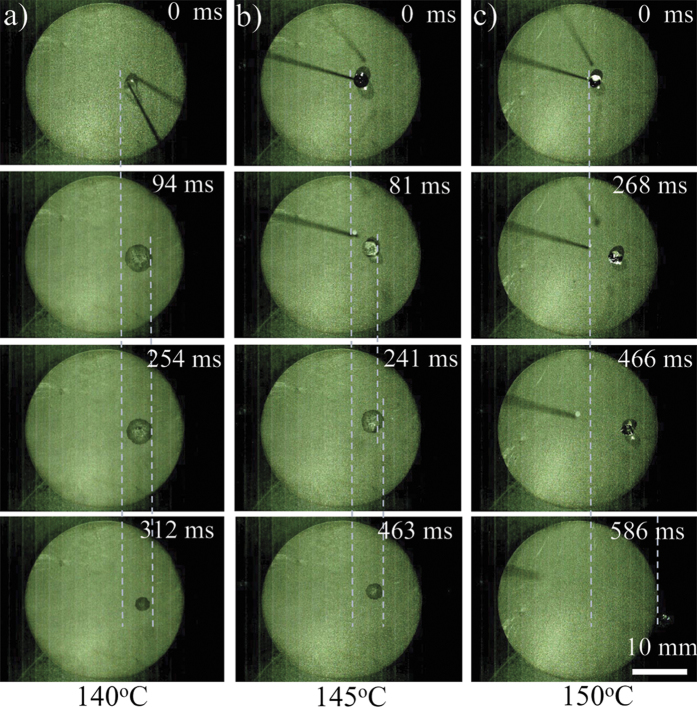
Movement behaviors of droplets on surface of Sample-A heated at different temperature. **a**) The movement behaviors of droplet on the surface at ~140 ^o^C. The droplet spreads along the wettable gradient direction at ~94 ms, and exhibits directional evaporation in ~94−312 ms. **b**) The movement behaviors of droplet on the surface at ~145^o^C. Initially, the droplet moves along the wettable gradient direction at ~81 ms and then exhibits directional spreading in ~81−241 ms. Finally, it exhibits directional evaporation in ~241−463 ms. **c**) The sliding behavior of droplet at ~150 ^o^C. The droplet keeps ball shape and slides off the horizontal surface fast along the wettable gradient direction at ~586 ms. The volume of droplet is 10 μL. The scale bar, 10 mm.

Figure 2 shows the movement behaviors of droplets on the surface of Sample-A heated at different temperatures. When the temperature varies from ~140 ^o^C to ~150 ^o^C, only nucleate boiling and gentle film boiling modes are observed. At ~140 ^o^C, since the temperature of the whole graphite plate is lower than Leidenfrost temperature ( ≥ 143.2 ^o^C), the boiling mode is nucleate boiling. When a droplet is dripped on the heated surface, it spreads along the wettable gradient direction at ~94 ms, exhibiting directional spreading and then evaporates extremely fast in ~94−312 ms, showing directional evaporation (Fig. 2a). At ~150 ^o^C, as the temperature is higher than Leidenfrost temperature ( ≤ 149.7 ^o^C), boiling mode changes to gentle film boiling on the whole surface. When a droplet is dripped on the heated surface, it keeps ball shape and slides off the horizontal surface fast along the wettable gradient direction only at ~586 ms (Fig. 2c). At ~145 ^o^C, initially, the droplet moves along the wettable gradient at 81 ms, just as that at ~150 °C because the temperature is larger than Leidenfrost temperature and the boiling mode is gentle film boiling. The increase of hydrophilicity induces the increase of Leidenfrost temperature. At the sites with the Leidenfrost temperature of above 145 °C, the boiling mode changes to nucleate boiling. The droplet exhibits directional spreading in ~81−241 ms and then directional evaporation in ~241 to ~463 ms (Fig. 2b), just as at ~140 ^o^C. The similar movement behaviors can also be observed on Sample-B. Clearly, not only the change of boiling mode, but the radial directional spreading, evaporation and sliding behaviors are observed in our work and the movement behaviors are controlled easily via temperature.

All the observations result from the nucleation, growth and departure of bubbles on wettable gradient surface. The bubble emission frequency is defined as^24^:


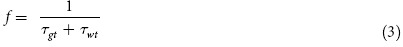


**Figure 3:**
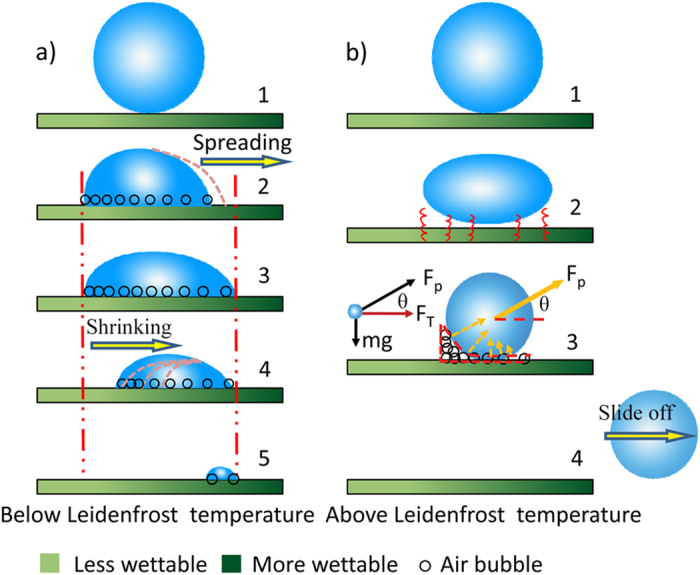
Schematic diagrams for the mechanism of directional droplet motion on over-heated wettable gradient surface. **a**) The directional spreading and evaporation behaviors of droplet on wettable gradient surface. The droplet initially spreads directionally along wettable gradient (Frame 2, 3). With lower superheat, the evaporation velocity of droplet at relative hydrophobic site is larger. The less wettable side shrinks and the more wettable side is pinned until the whole droplet disappears, exhibiting directional evaporation (Frame 4, 5). **b**) The directional sliding behaviors of droplets on wettable gradient surface with gentle film boiling mode. At higher superheat, the decrease of waiting time induces a larger bubble emission frequency and the generated bubbles are easier to merge to vapor blanket (Frame 2). It induces the droplet in a ball shape (Frame 3). More bubbles at the hydrophobic site induce vapor pressure gradient. Droplets slide off the graphite plate quickly under effects of pressure and gravity (Frame 4).

Here *f* is the bubble emission frequency,τ_*gt*_ is the growth time (the duration of the growth of bubbles) and τ_*wt*_ is the waiting time (the duration between the departure of the former bubble and the appearance of the current bubble). Hai Trieu Phan *et al.*^24^ demonstrated that the bubble emission frequency decreased with the rise of surface hydrophilicity and the decrease of temperature. At low superheat (below Leidenfrost temperature, ~140 ^o^C), the boiling mode is nucleate boiling. Due to the fact that the drop tends to create nearly equal contact angles at both edges under effect of the Laplace pressure within the drop^25^ and the unbalanced wettability force points to the more wettable side, the droplet initially spreads along wettable gradient, exhibiting directional spreading (Fig. 3a, Frame 2, 3). Subsequently, since the hydrophobic surface performs more favorable conditions for the formation and growth of bubbles than hydrophilic surface (the evaporation velocity on relative hydrophobic sites is larger) and wettability force points to the more wettable side, the less wettable side shrinks and the more wettable side is pinned until the whole droplet disappears, exhibiting directional evaporation (Fig. 3a, Frame 4, 5). At high superheat (above Leidenfrost temperature), the boiling mode is gentle film boiling. The decrease of waiting time induces a larger bubble emission frequency and the generated bubbles are easier to merge to vapor blanket (Fig. 3b, Frame 2). So, the droplet would keep a ball shape (Fig. 3b, Frame 3). More bubbles are generated at the hydrophobic site and a vapor pressure gradient is introduced. Due to the high pressure at the hydrophobic site, the total pressure could be written as *F*_*p*_, which points to the more wettable side and has an angle *θ* with horizontal plane. Under the effect of pressure and gravity (mg) (m is the mass of droplet, g is the acceleration due to gravity), a horizontal total force *F*_*T*_ is generated. Due to the Leidenfrost effect^17^, hysteresis force (*F*_*H*_) becomes much smaller than total force *F*_*T*_. Therefore, above Leidenfrost temperature, a droplet keeps ball shape with high CA and slides off the horizontal surface fast along the wettable gradient direction (Fig. 3b, Frame 4), corresponding to the observation at ~150 ^o^C. At ~145 ^o^C, due to the different boiling modes on the different sites, all of the directional spreading, evaporation and movement are observed.

**Figure 4:**
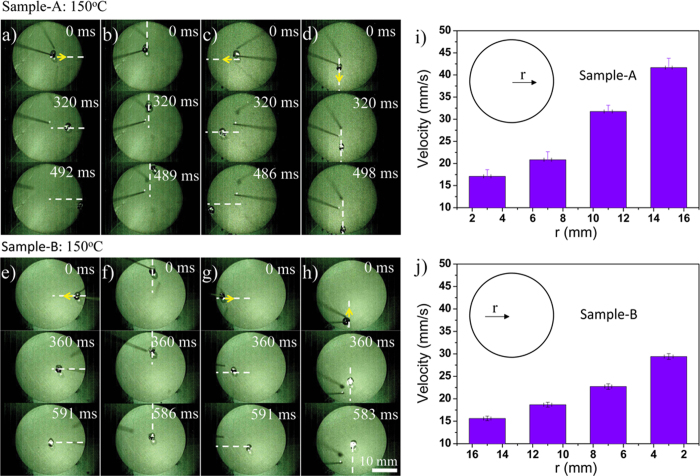
Outside radial movement of droplet on surface of Sample-A at ~150 ^o^C. **a**) Droplet moves from center to right periphery. **b**) Droplet moves from center to top periphery. **c**) Droplet moves from center to left periphery. **d**) Droplet moves from center to bottom periphery. When the droplet is dripped beside the center of the surface, it slides along the outside radial direction (i.e., wettable gradient direction) and sliding off the surface in less than ~500 ms. **Inside radial movement of droplet on surface of Sample-B** at **~150**^**o**^**C. e**) Droplet moves from above periphery to center. **f**) Droplet moves from left periphery to center. **g**) Droplet moves from right periphery to center. **h**) Droplet moves from bottom periphery to center. When the droplet is dripped at the periphery of the Sample-B surface, it could slide along the inside radial direction (i.e., wettable gradient direction) and move to the center of graphite plate in less than 600 ms. **i**) Sliding velocities of droplets on different points on surface of Sample-A at ~150 ^o^C. **j**) Sliding velocities of droplets on different points on surface of Sample-B at ~150 ^o^C (*r* is the distance to center of graphite plate). The volume of the droplet is 10 μL. The scale bar, 10 mm.

We surveyed the directional movement behaviors of droplets on different points of the graphite plates on Sample-A and Sample-B at ~150 ^o^C. For Sample-A, when a droplet is dripped near to the center of the surface, it can slide along the outside radial direction (i.e., wettable gradient direction) and slide off the surface in less than ~500 ms with a velocity of ~ 40 mm/s (Fig. 4 a-d,i). Otherwise, when a droplet is dripped at the periphery of the Sample-B surface, it could also slide along the inside radial direction (i.e., wettable gradient direction) with the velocity of ~ 30 mm/s (Fig. 4 e-h,j). After the droplet passes the center, the direction of wettable gradient force reverses, which would hinder the movement of droplet. Therefore, the velocity slows down gradually until the droplet slides off the surface (Figure S8). Clearly, controlled radial spreading or convergence and divergence of droplets on the graphite plate at high temperature are realized for the first time by a one-step anodic oxidation method.

In conclusion, radial wettable gradient with direction toward the periphery or the centre of graphite plate was easily achieved by a one-step anodic oxidation method. At room-temperature, droplets on such kind of surface could exhibit controlled radial spreading behaviors. At higher temperature, due to the wettable gradient, not only the transition of boiling mode, but also the directional spreading, evaporation and movement behaviors of the droplets were observed. The radial convergence and divergence of droplets were controlled effectively on the graphite plate at high temperature, which can be extended to realm of micro-fluidics and the exploration of the biotechnology.

## Methods

### Preparation of a circular graphite plate with radial wettable gradient

A circular graphite plate (diameter: 30 mm, thickness: 3 mm, manufactured by Ji Xing Sheng, in Co. of Beijing, China) with no visible scratches were rinsed thoroughly with ethanol and deionized water for following use. The radial wettable gradient on a circular graphite plate was fabricated by an improved anodic oxidation with 0.05 M sodium hydrate (NaOH) electrolyte solution and a constant current of 0.5 A. The electrolyte level is set above or below the center of the circular graphite plate with a certain distance (*L*) to control the direction of wettable gradient. During the anodic oxidation process, the circular graphite plate was rotated uniformly for a cycle with a constant period (*T*). The schematic diagram of experimental set-up was shown in Fig. 1a,c. The center of circular graphite plate is zero point position.

### Characterization

Water contact angles (CAs) were measured by the optical contact angle meter system (OCA40Micro, Dataphysics Instruments GmbH, Germany) and the static CAs were determined by the averages of at least five measurements. The volume of the droplet is 5 μL. The directional movement behaviors of droplets were observed by a high speed CCD (HSCCD, V9.1, PHANTOM, America). The temperature was controlled by heating stage (IKA C-MAG HS7). Scanning electron microscope (SEM, Quanta FEG 250, FEI) was employed for the surface micro-structure analysis of graphite plate. The chemical composition was measured by field emission scanning electron microscopy (FESEM) with energy dispersive spectrometer (EDS) (JSM-7500 F, JEOL, Japan).

## Additional Information

**How to cite this article**: Feng, S. *et al*. Radial Wettable Gradient of Hot Surface to Control Droplets Movement in Directions. *Sci. Rep.* **5**, 10067; doi: 10.1038/srep10067 (2015).

## Supplementary Material

Supplementary Information
