# Supplementary material for: Radial Wettable Gradient of Hot Surface to Control Droplets Movement in Directions
Source: Sci Rep. 2015 May 15;5:10067. doi: 10.1038/srep10067 (PMC4432314; doi:10.1038/srep10067)
Supplement: Supplementary Information [file srep10067-s1.pdf]

Supplementary Information

Radial Wettable Gradient of Hot Surface to Control Droplets Movement in Directions

Shile Feng, Sijie Wang, Yuanhao Tao, Weifeng Shang, Siyan Deng, Yongmei Zheng, Yongping Hou*.*

[*]Prof. Y. Zheng, Dr. Y. Hou, Dr. S. Feng, S. Wang, Y. Tao, W. Shang, S. Deng

Key Laboratory of Bio-Inspired Smart Interfacial Science and Technology of Ministry of Education, Beijing Key Laboratory of Bio-inspired Energy Materials and Devices School of Chemistry and Environment
Beihang University
Beijing, 100191 (P. R. China)
E-mail: zhengym@buaa.edu.cn; houyongping09@buaa.edu.cn

Content：

Supplementary Figure Legends: Figure S1-S8

Supplementary Table: Table S1

Supplementary Figure Legends：

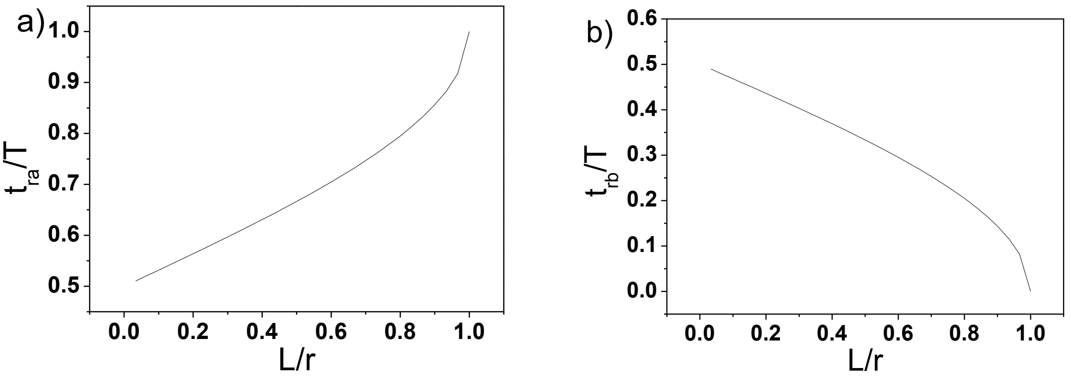

Figure S1 | Anodic oxidation time at the different points for different conditions. **a**) When electrolyte level is blow the center for a constant distance, the oxidation time (t_{rb}) increases gradually with the increase of the distance *r*. **b**) When electrolyte level is above the center for a constant distance, the oxidation time (t_{ra}) increases gradually with the decrease of the distance *r* (*L* is the distance between electrolyte level and the center of graphite plate. *T* is the rotation period. *r* is the distance to the center of graphite plate).

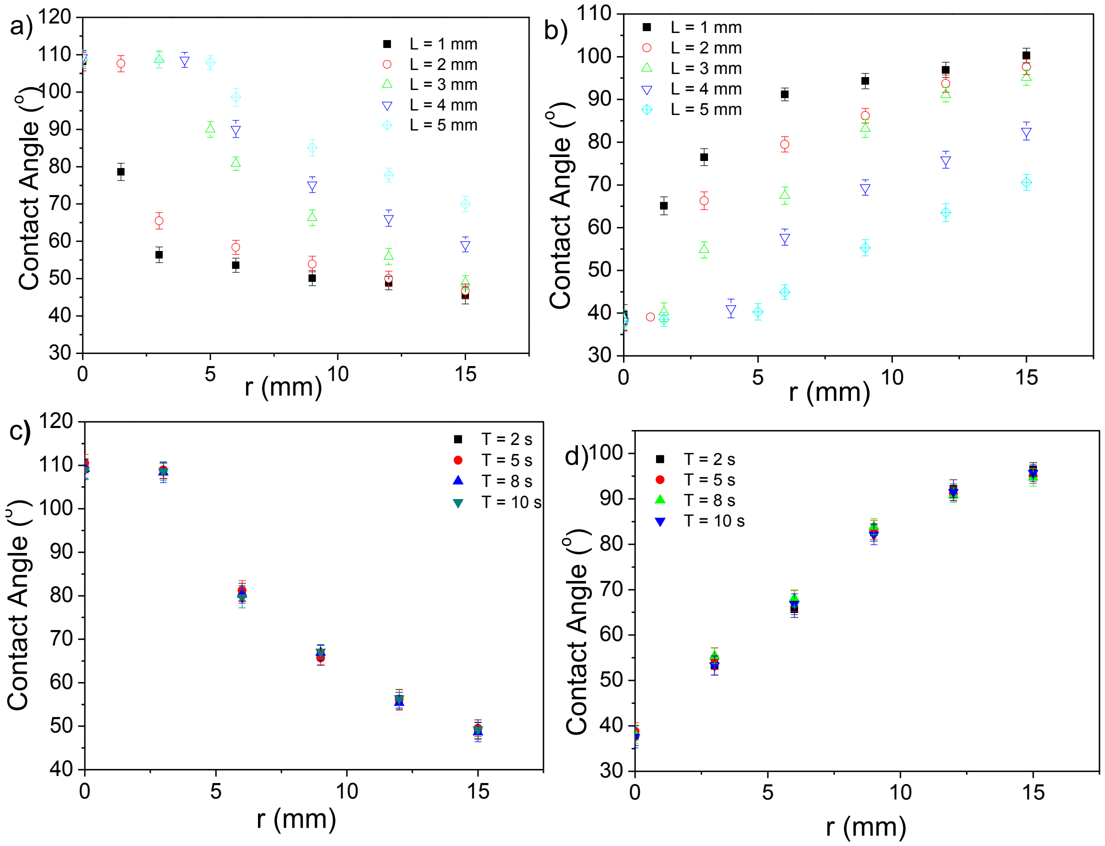

Figure S2 | Influences of the value of *L* and *T* on the wettable gradient. a) The influence of *L* on the wettable gradient surface of Sample-A. The current is 0.5 A and the period of rotation is 5 s. **b**) The influence of *L* on the wettable gradient surface of Sample-B. The current is 0.5 A and the period of rotation is 5 s. **c**) The influence of *T* on the wettable gradient surface of Sample-A. The current is 0.5 A and the *L* is 3 mm. d) The influence of *T* on the wettable gradient surface of Sample-B. The current is 0.5 A and the *L* is 3 mm. The radial wettable gradient on a circular graphite plate could be easily fabricated by an improved anodic oxidation method and the values of wettable gradient could be controlled via the two parameters, e.g., *L* and *T* (*L* is the distance between electrolyte level and the center of graphite plate. *T* is the rotation period. *r* is the distance to the center of graphite plate).

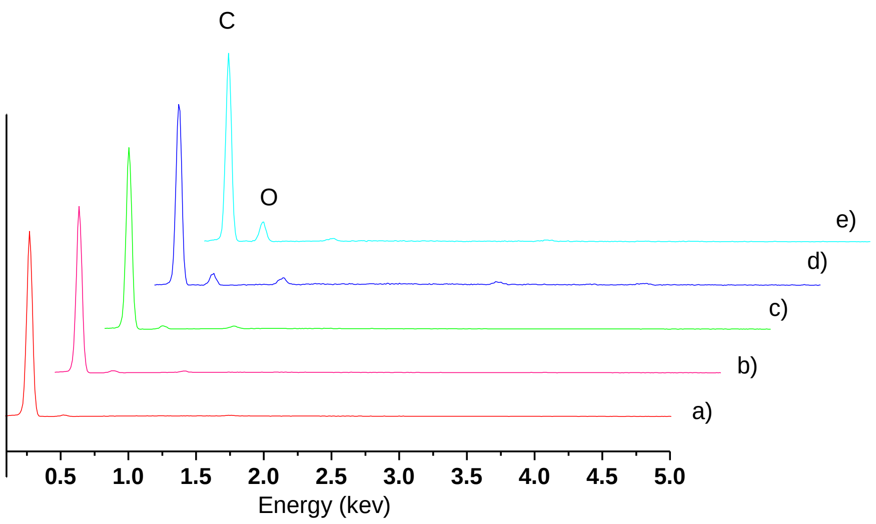

Figure S3 | EDS curves of of different points on Sample-A. a) 0 mm, **b**) 3 mm, **c**) 7 mm, **d**) 11 mm, **e**) 15 mm (distance to the center).

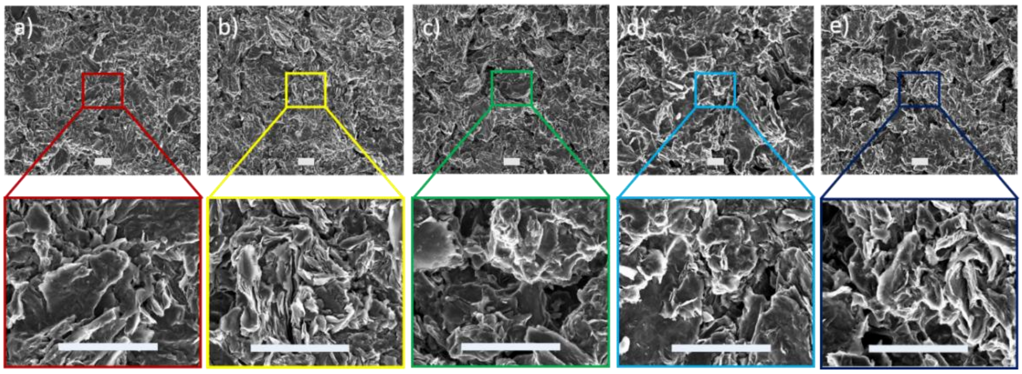

Figure S4 | SEM images of different areas on Sample-A. a) 0 mm, **b**) 3 mm, **c**) 7 mm, **d**) 11 mm, **e**) 15 mm (distance to the center). The scale bars are 30 μm.

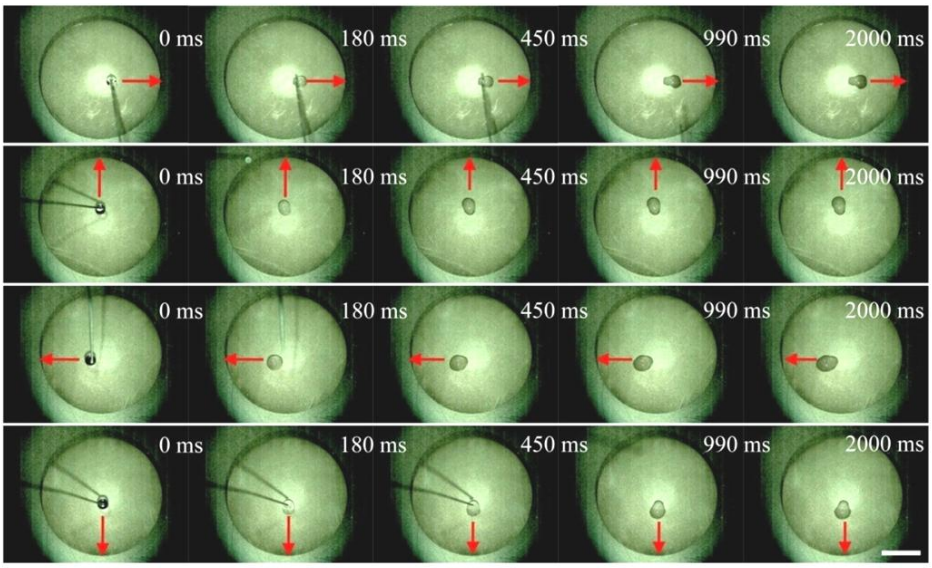

Figure S5 | Directional spreading behaviors of droplets on the surface of Sample-A at room-temperature. The volume of the droplet is 10 μL, and the scale bar is 10 mm.

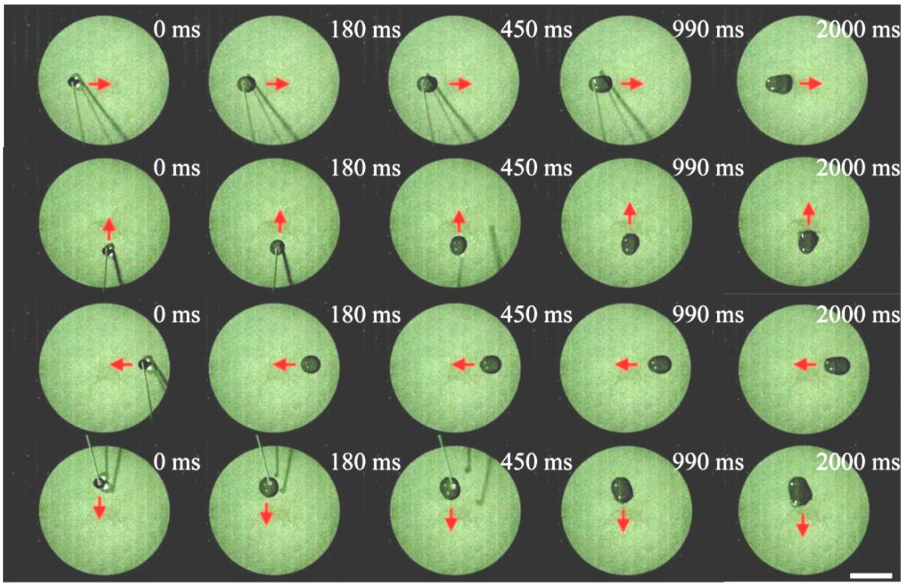

Figure S6 | Directional spreading behaviors of droplets on the surface of Sample-B at room-temperature. The volume of the droplet is 10 μL, and the scale bar is 10 mm.

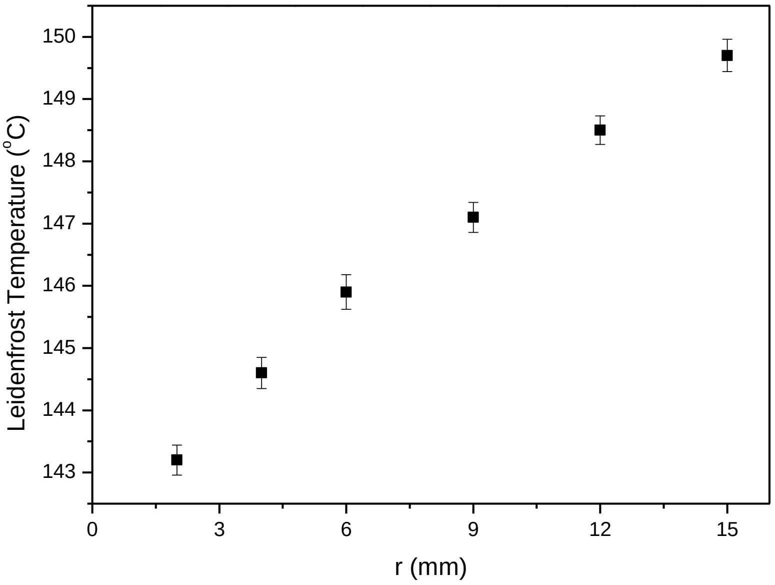

Figure S7 | Leidenfrost temperature at the different points on heated Sample-A (*r* is the distance to the center of graphite center).

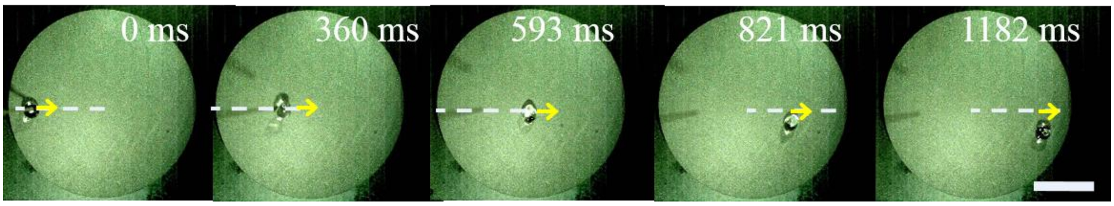

Figure S8 | Movement behaviors of droplets on wettable gradient surface of Sample-B. The volume of the droplet is 10 μL and the scale bar is 10 mm.

Table S1 | O, C and O/C atomic ratios of different points on Sample-A.

Distance/ mm	0	3	7	11	15
O / %	4.28	6.86	9.93	15.63	25.55
C / %	95.58	92.63	89.28	81.86	73.69
O /C	0.0448	0.0721	0.1112	0.1909	0.3467
